# Targeting the Interplay Between Cancer Fibroblasts, Mesenchymal Stem Cells, and Cancer Stem Cells in Desmoplastic Cancers

**DOI:** 10.3389/fonc.2019.00688

**Published:** 2019-07-31

**Authors:** Tze-Sian Chan, Yuval Shaked, Kelvin K. Tsai

**Affiliations:** ^1^Laboratory of Advanced Molecular Therapeutics, Wan Fang Hospital, Taipei Medical University, Taipei, Taiwan; ^2^Division of Gastroenterology, Department of Internal Medicine, Wan Fang Hospital, Taipei Medical University, Taipei, Taiwan; ^3^Integrative Therapy Center for Gastroenterologic Cancers, Wan Fang Hospital, Taipei Medical University, Taipei, Taiwan; ^4^School of Medicine, College of Medicine, Taipei Medical University, Taipei, Taiwan; ^5^Department of Cell Biology and Cancer Science, Rappaport Faculty of Medicine, Technion – Israel Institute of Technology, Haifa, Israel; ^6^Technion Integrated Cancer Center, Technion – Israel Institute of Technology, Haifa, Israel; ^7^Graduate Institute of Clinical Medicine, College of Medicine, Taipei Medical University, Taipei, Taiwan; ^8^National Institute of Cancer Research, National Health Research Institutes, Taipei, Taiwan

**Keywords:** cancer-associated fibroblasts, mesenchymal stem cells, cancer stem cells, paracrine signaling, desmoplasia

## Abstract

Malignant tumors are highly heterogeneous and likely contain a subset of cancer cells termed cancer stem cells (CSCs). CSCs exist in a dynamic equilibrium with their microenvironments and the CSC phenotype is tightly regulated by both cell-intrinsic and cell-extrinsic factors including those derived from their surrounding cells or stroma. Many human solid tumors like breast, lung, colorectal and pancreatic cancers are characterized by a pronounced stromal reaction termed “the desmoplastic response.” Carcinoma-associated fibroblasts (CAFs) derived either from resident fibroblasts or tumor-infiltrating mesenchymal stem cells (MSCs) are a major component of the stroma in desmoplastic cancers. Recent studies identified subpopulations of CAFs proficient in secreting a plethora of factors to foster CSCs, tumor growth, and invasion. In addition, cytotoxic therapy can lead to the enrichment of functionally perturbed CAFs, which are endowed with additional capabilities to enhance cancer stemness, leading to treatment resistance and tumor aggressiveness. When recruited into the tumor stroma, bone-marrow-derived MSCs can promote cancer stemness by secreting a specific set of paracrine factors or converting into pro-stemness CAFs. Thus, blockade of the crosstalk of pro-stemness CAFs and MSCs with CSCs may provide a new avenue to improving the therapeutic outcome of desmoplastic tumors. This up-to-date, in-depth and balanced review describes the recent progress in understanding the pro-stemness roles of CAFs and tumor-infiltrating MSCs and the associated paracrine signaling processes. We emphasize the effects of systemic chemotherapy on the CAF/MSC–CSC interplay. We summarize various promising and novel approaches in mitigating the stimulatory effect of CAFs or MSCs on CSCs that have shown efficacies in preclinical models of desmoplastic tumors and highlight the unique advantages of CAF- or MSC-targeted therapies. We also discuss potential challenges in the clinical development of CSC- or MSC-targeted therapies and propose CAF-related biomarkers that can guide the next-generation clinical studies.

## Introduction

### Cancer Stem Cells (CSCs) as the Driving Force of Tumor Progression

An emerging concept of cancer biology emphasizes the critical role of the hierarchical organization in tumors in the maintenance as well as the progression of the malignant phenotypes. In support of this paradigm, mounting data over recent years, including large-scale genomic analysis and single-cell RNA sequencing analysis, have consistently indicated the existence of a subset of cancer cells termed the tumor-initiating cells (TICs) or CSCs, which are stem-like and have the capability of self-renewing and sustaining tumorigenesis and thereby serve as the driving force of cancer growth, metastasis, and treatment resistance (1–3). CSCs have been found to exist in leukemia and multiple solid tumors, such as glioma, breast cancer, pancreatic ductal adenocarcinoma (PDAC), head and neck squamous cell carcinoma, hepatocellular carcinoma, non-small cell lung cancer (NSCLC), and colorectal cancer (CRC) (4–8).

The recent insights into the complex nature of cancer stemness reveal that CSCs exist in a dynamic equilibrium with their microenvironments and the CSC phenotype is regulated by both cell-intrinsic and cell-extrinsic factors derived by their surrounding cells or stroma cells. The notable examples of the “pro-stemness” or “pro-CSC” factors identified from these studies are inflammatory cytokines, such as interleukin-6 (IL-6), IL-8, and C-C motif chemokine ligand 5 (CCL-5), which play an essential role in CSC regulation as well as invasion and metastasis of tumors (9–11).

### CAFs and MSCs Foster Cancer Stemness

Many types of human solid tumors, especially those derived from glandular epithelium, such as breast cancer, NSCLC, PDAC, the scirrhous subtype of gastric adenocarcinoma, and the “stem/serrated/mesenchymal (SSM)” molecular subtype of CRC, are characterized by a pronounced stromal reaction termed “the desmoplastic response” (12–17). CAFs and their collagen matrix products are a major component of the stroma in desmoplastic cancers, comprising a substantial proportion of the tumor mass (18, 19). Instead of being functional inert, there is circumstantial evidence that CAFs are pro-inflammatory due to activation of nuclear factor kappa B (NF-κB), signal transducer and activator of transcription (STAT)-1 and−3, and transforming growth factor (TGF)-β/SMAD signaling and are engaged in active cross-talk with cancer cells (19, 20). Therefore, CAFs can foster tumor cell growth, angiogenesis and invasion (21) by secreting paracrine factors, such as pro-inflammatory cytokines (19), chemokines (14, 19), prostaglandins (PGE) (22), growth factors (23), and proteases (24), and by remodeling the extracellular matrix (ECM) (25–28). CAFs also help foster an immunosuppressive microenvironment in tumors by promoting regulatory T cells (29). Recent studies demonstrated that exosomes derived from CAFs promote cancer progression and treatment resistance (30, 31). Intriguingly, CAFs can even travel with malignant cells to distant sites, where they significantly promote metastasis (32). One of the major mechanisms by which CAFs promote oncogenesis is mediated through their pro-stemness abilities. Recent studies have identified specific subpopulations of CAFs that are proficient in secreting pro-stemness paracrine factors (9–11, 23, 33–35), thereby promoting the conversion of cancer cells into CSCs or supporting the self-renewal and the stemness properties of existing CSCs in tumors. Upon stimulation by cytotoxic stress such as chemotherapy, CAFs can be further induced to secrete pro-stemness cytokines or acquire a senescence-like secretory phenotype and produce large amounts of pro-stemness chemokines to further enhance tumor stemness and aggressiveness following therapy (36, 37).

Although the majority of CAFs in the tumor stroma may be derived from resident stromal fibroblasts, there are now multiple lines of evidence suggesting that a significant proportion of CAFs in tumors are derived from bone marrow-derived mesenchymal stem cells (MSCs). MSCs are pluripotent stem cells that contribute to bone, adipose, cartilage, and muscle tissues and are involved in tissue remodeling, chronic inflammation, immune response, and cancer progression (38). Bone marrow-derived MSCs can be recruited to sites of tissue damages or inflammation by endocrinal signals to exert their tissue repairing functions (39), whereas the tissue-regenerative function of MSCs may go awry in malignant tumors. For instance, in mouse models of breast cancer, PDAC or gastric cancer, bone marrow-derived MSCs are recruited to the tumor microenvironment where they differentiate into CAFs (40–42). Indeed, in a gastric cancer model, approximately 20% of CAFs were found to originate from bone marrow-derived MSCs, which were recruited into the tumors in a transforming growth factor (TGF)-β and C-X-C motif chemokine ligand (CXCL)-12-dependent manner (43). Similarly, MSCs introduced into the tibia trafficked to sites of breast tumor xenografts (44). In an orthotopic murine PDAC model, MSCs were actively recruited into the growing pancreatic tumors (45). Like CAFs, MSCs can significantly influence tumor behaviors and contribute to tumor progression. Most importantly, MSCs promote CSCs by secreting a plethora of pro-stemness cytokines and growth factors or indirectly by differentiating into pro-stemness CAFs (44, 46, 47).

### Cancer Therapy Can Alter Tumor Stroma and Promote Tumor Stemness

In clinical scenarios, most cancers are treated with certain types of cytotoxic therapies, such as chemotherapy and radiation therapy, which may have profound impacts on the characteristics of tumors including the epithelial and the stromal compartments. Indeed, chemotherapy has been shown to enrich tumor cells for those with mesenchymal and/or CSC features in different types of cancers. CSCs are intrinsically more resistant to therapy and consequently increase disproportionately following systemic chemotherapy and are thought to contribute to tumor relapse and treatment resistance (1, 48, 49). For instance, breast cancers after neoadjuvant chemotherapy are enriched from CD44^+^CD24^−^ CSCs that also express mesenchymal markers (48, 49). Chronic oxaliplatin or paclitaxel treatment induces an epithelial-mesenchymal transition (EMT) and the enrichment of CSCs in CRC and ovarian cancer (50, 51). Chemotherapy has also been shown to expand CSCs that are dependent on the interleukin (IL)-8–CXCR-1 signaling axis (52). Importantly, CAFs are enriched in chemotherapy-treated human tumor tissues wherein they promote cancer growth, treatment resistance and the self-renewal of CSCs by secreting paracrine factors (36, 53). Moreover, chemotherapy-modulated CAFs secrete a panel of CXCL chemokines to expand CSCs in the treated tumor, leading to paradoxical tumor aggression and treatment failure (37). Thus, adjuvant strategies that target CAFs to temper the chemotherapy-induced enrichment of CSCs may further improve the therapeutic outcome of patients with desmoplastic cancers.

The past two decades of investigations into CSCs and their biology have led to the identification of a number of potentially druggable targets, based on which many CSC-directed therapies have been developed with some of them entering clinical trials (54). Unfortunately, the idea of therapeutic targeting of CSCs has suffered from a series of notable clinical trial failures over recent years, including the focal adhesion kinase (FAK) inhibitor defactinib, the STAT-3 inhibitor napabucasion, the anti-NOTCH-2/3 antibody tarextumab, the anti-delta like canonical notch ligand (DLL)-4 antibody demcizumab, and most recently the multibillion-dollar anti-DLL-3 antibody-drug conjugate rovalpituzumab tesirine (Rova-T). Apparently, there is an urgent need for new and more viable strategies of successfully and safely targeting CSCs. As opposed to the direct targeting of the rare, dynamic and plastic CSC populations, targeting the more abundant, favorably spaced and stable CAFs and MSCs, especially their pro-stemness subsets, presents an attractive strategy to indirectly target cancer stemness to enhance the efficacy of current anti-cancer therapies.

In this review, we describe how CAFs and MSCs initiate crosstalk with CSCs and augment cancer stemness in human solid tumors. We emphasize the effects of systemic chemotherapy on CAFs and how these effects can modulate their pro-stemness functions in the treated tumor. We discuss the advantages of targeting CAFs or MSCs over directly targeting CSCs, as well as various promising approaches that aim at disengaging the CAF/MSC–CSC link in preclinical models. This review finally lists potential challenges in the clinical development of pro-stemness-CAF- or MSC-targeted therapies and explores potential biomarkers of pro-stemness CAFs to guide the development of therapeutic strategies to disengage the dangerous interplay between CAFs, MSCs, and CSCs that can be quickly deployed in clinical trials in the treatment of human desmoplastic cancers.

## CAFs and CSCs in Desmoplastic Cancers: the Mesenchymal–Epithelial Crosstalk Goes Awry

**Figure 1:**
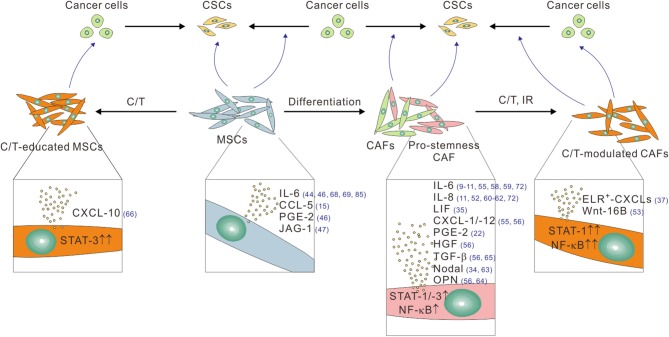
The pro-stemness functions of CAFs and tumor infiltrating MSCs linked to their different functional and treatment status. CAFs, especially their pro-stemness subset, secrete assorted cytokine and chemokines, including IL-6, IL-8, LIF, PGE-2, CXCL-1, CXCL-12, HGF, and TGF-β through heightened STAT, and NF-κB signaling activity to promote the reprogramming of cancer cells into CSCs and/or directly expand the CSC population. CAFs also secret Nodal and osteopontin (OPN) to promote CSCs. Cytotoxic therapies such as chemotherapy (C/T) and ionizing radiation (IR) further potentiate the pro-stemness functions of CAFs by further activating STAT-1 and NF-κB signaling, thereby inducing the secretion of a different panel of pro-stemness factors, including ELR^+^-CXCL chemokines, and Wnt-16B. Bone marrow-derived and tumor-infiltrating MSCs can promote CSCs by converting into CAFs or by secreting several pro-stemness chemokines such as IL-6, CCL-5, PGE-2, and JAG-1. C/T-educated MSCs further secrete the pro-stemness chemokine CXCL-10 by activating STAT-1 signaling. The reference numbers are shown in blue.

As described above, CAFs are proficient in paracrine signaling and are capable of secreting a plethora of paracrine factors that have been implicated in the maintenance and/or the expansion of CSCs (Figure 1 and Table 1). Among the most extensively studied pro-stemness cytokines secreted by CAFs are IL-6 and IL-8, which have been shown to play an essential role in the regulation of CSCs as well as cancer invasion and metastasis (9–11). Several mechanistic studies have demonstrated that IL-6 participates in the regulation and maintenance of the CSC phenotype mainly through the STAT-3–NF-κB signaling pathway (10, 11, 57). Constitutive IL-6 expression in breast cancer cells maintains their EMT phenotype, which has been implicated in the generation of a CSC phenotype (58, 59). As opposed to the role of the IL-6 inflammatory loop in inducing CSCs with mesenchymal features in breast cancer, IL-8 mainly regulates a subpopulation of epithelial-like CSCs that express high aldehyde dehydrogenase (ALDH) activity and are highly proliferative (52). Consistently, IL-8 was found to profoundly enhance the stemness property of breast cancer and PDAC cells (60–62). A recent proteomic screening identified leukemia inhibitory factor (LIF)-induced STAT-3 activation as the major signaling event in PDAC cells induced by PSCs, leading to activation of stemness programs, including Hippo, Wnt, and STAT-3 (35). Notably, LIF expression is significantly up-regulated in PDAC tissues while the expression of IL-6 does not, underscoring the importance of LIF over IL-6 in PDAC. Aside from interleukins, a multitude of other secretory factors has also been implicated in mediating the pro-stemness capability of CAFs. For instance, in CRC models established using primary carcinoma cells, CAF-derived osteopontin (OPN) has been shown to support the clonogenic capacity of CSCs, which predominantly reside at the tumor edge in close proximity to CAFs (64). Another study also showed that the CAFs freshly isolated from human CRC tumors produced significantly higher levels of CXCL-12, OPN, TGF-β, and hepatocyte growth factor (HGF), which coordinately activated Wnt–β-catenin signaling to induce the expression of the novel CSC marker CD44 variant 6, resulting in an EMT in cancer cells and tumor invasion and metastasis (56). In another CRC model established using freshly isolated carcinoma cells and the paired CAFs, CAFs up-regulated the expression of TGF-β2 and IL-6, which activated the expression of GLI family zinc finger (GLI)-2 in the sonic hedgehog (SHH) pathway, resulting in the transdifferentiation of cancer cells into CSCs and chemotherapy resistance (65). Pancreatic stellate cells (PSCs), a specialized type of CAFs present in the stroma of PDAC, secrete the TGF-β family protein Nodal, which binds to its receptor Activin-like (Alk)-4 and−7 on CSCs to promote their stemness properties (34, 63). In an NSCLC model, insulin-like growth factor (IGF)-II and allied autocrine/paracrine factors secreted by CAFs synergistically activated IGF-1R signaling to induce the expression of the stemness-related gene Nanog, thereby converting cancer cells into CSCs (23). CAFs isolated from human breast cancer secrete abundant levels of PGE-2, which enhances the secretion of IL-6 to expand CSCs (22). Moreover, when co-cultivated with cancer cells, CAFs produced a higher level of CCL-2, which stimulate CSCs by inducing Notch-1 expression and thereby activating the Notch signaling pathway (33).

**Table 1:** Pro-stemness factors secreted by CAFs and MSCs.

**Factors**	**Cancer types**	**Functions**	**Stemness pathway involved**	**References**
**CAFs**				
CCL-2	BC	Stimulates CSCs by inducing Notch-1 expression	Notch-1	(33)
CXCL-1	PDAC	Promotes cancer stemness	IL-1α/JAK/STAT	(55)
CXCL-12	CRC	Induces the expression of CSC markers	Wnt/CD44v6, PI3K	(56)
CXCL-2	PDAC	Promotes cancer stemness	IL-1α/JAK/STAT	(55)
ELR^+^ CXCLs	BC, PDAC	Secreted by chemotherapy-altered CAFs and promote CSC expansion	STAT-1, NF-κB	(37)
HGF	CRC	Induces the expression of CSC markers	PI3K	(56)
IGF-II	NSCLC	Induces conversion of cancer cells into CSCs	IGF1R, EMT, PI3K, TGF-β, Wnt, and Hedgehog	(23)
IL-17A	CRC	Promotes the self-renewal of CSCs and tumor growth		(36)
IL-6	BC	Promotes and maintains CSCs	STAT-3 and NF-κB	(10, 11, 57)
	BC	Maintains EMT phenotype and stem cell properties	EMT	(58, 59)
	PDAC	Pro-stemness factor	IL-1α/JAK/STAT	(55)
IL-8	BC	Promotes epithelial-like ALDH^+^ CSCs	FAK/AKT/FOXO-3A	(52)
	BC, PDAC	Enhances stemness property		(60–62)
LIF	PDAC	Activates stemness program, including Hippo, Wnt and STAT-3	STAT-3	(35)
Nodal	PDAC	Binds to Alk-4/-7 to promote stemness in cancer cells	Nodal/activin	(34, 63)
OPN	CRC	Supports the clonogenic capacity of CSCs	Wnt/CD44v6, PI3K	(56, 64)
		Induces expression of CSC marker		
PGE-2	BC	Promotes secretion of IL-6 and expansion of CSCs	NF-κB	(22)
TGF-β	CRC	Induces the expression of CSC marker	Wnt/CD44v6, PI3K	(56)
TGF-β2	CRC	Induces trans-differentiation of cancer cells into CSCs and confers chemo-resistance	Hedgehog/GLI-2	(65)
WNT16B	PC	Enriches CSCs and promotes proliferation and invasion of cancer cells	Wnt, EMT	(53)
**MSCs**				
CCL-5	BC	Promotes cancer stemness and tumor metastasis		(15)
CXCL-10	PDAC	Promotes cancer stemness and expand the number of MSCs.	CXCR-3	(66)
CXCL-3	PDAC	Promote CSCs following gemcitabine therapy	STAT-3/CXCR-3	(67)
CXCL-7	BC	Promotes CSCs and tumor growth	IL-6	(68)
IL-6	BC	Regulates CSCs and promotes tumor growth	β-catenin	(46)
	CRC	Promotes drug resistance following paclitaxel therapy	JAK-2/STAT-3	(69)
JAG-1	PDAC	Maintains CSCs	Notch-1	(47)
PGE-2	CRC	Induces the formation of CSCs by inducing the expressions of IL-6, IL-8, and CXCL-1.	Wnt	(46)
PUFA	BC, CRC, LC	Promotes the regrowth of tumors following chemotherapy	Cyclooxygenase-1/thromboxane synthase	(70)

*Alk-4, activin-like 4; BC, breast cancer; CCL, chemokine C-C motif ligand; CRC, colorectal cancer; CXCL, C-X-C motif chemokine ligand; ELR^+^ CXCLs, ELR motif–positive chemokines; EMT, epithelial-mesenchymal transition; FAK, focal adhesion kinase; FOXO-3A, Forkhead box O3; GLI-2, GLI family zinc finger 2; HER-2, human epidermal growth factor receptor 2; HGF, hepatocyte growth factor; IGF, insulin growth factor; IL, interleukin; JAG, Jagged; JAK, Janus kinase; LC, lung carcinoma; LIF, leukemia inhibitory factor; NF-κB, nuclear factor kappa-light-chain-enhancer of activated B cells; NSCLC, non-small cell lung cancer; OPN, osteopontin; PC, prostate cancer; PDAC, pancreatic ductal adenocarcinoma; PGE, prostaglandin; PI3K, phosphatidylinositol 3-kinase; PUFA, polyunsaturated fatty acids; STAT, signal transducer and activator of transcription; TGF, transforming growth factor*.

Mounting data accumulated over recent years have suggested that CAFs in desmoplastic cancers are phenotypically, functionally and genetically heterogeneous and are likely dynamically controlled by their environments and origins (29, 71–76). Indeed, CAFs have been classified into various functional subtypes according to a panel of surface markers, including such as α-SMA, fibroblast activation protein (FAP), fibroblast specific protein (FSP)-1, and platelet-derived growth factor receptor (PDGFR)-α/β (19, 77, 78). At the functional level, FAP^+^ CAFs are enriched in low stiffness and fibronectin-rich ECMs, whereas α-SMA^+^ CAFs are found in stiffer ECM contexts (76). In a transgenic model of PDAC, depletion of α-SMA^+^ CAFs did not affect the number of FAP^+^ CAFs, indicating that they represent different CAF subpopulations (79). Interestingly, FAP^+^ CAFs are predominantly involved in the synthesis and the turnover of ECM while α-SMA^+^ CAFs mediate contraction. Importantly, the recently identified CAF heterogeneity relates to the pro-stemness and pro-oncogenic capabilities of CAFs. For instance, in oral squamous cell carcinoma, a subgroup of CAFs termed “CAF-D” has been shown to induce EMT of malignant keratinocytes through secreting TGF-β (74). Since the EMT program in cancer cells imparts them with CSC features (59, 80, 81), it is likely that this specific subpopulation of CAFs might have induced the phenotypic conversion of keratinocytes into CSCs. In PDAC, two distinct subgroups of PSCs have been identified in mouse and human PDAC tissues (71). Remarkably, only those PSCs located away from tumor cells, denoted as “inflammatory CAFs (iCAFs),” were proficient in secreting pro-stemness factors, including IL-6, CXCL-1, and CXCL-2, through activation of IL-1α-Janus kina (JAK)–STAT signaling (55). By contrast, PSCs located adjacent to tumor cells have the propensity of differentiating into collagen-producing and α-smooth muscle actin (α-SMA)-positive myofibroblasts. In analogous to this emerging paradigm of the functional heterogeneity of CAFs, in human breast cancer and NSCLC tissues, a distinct subpopulation of CAFs was found to express CD10 as well as the complement 5 a receptor G-protein coupled receptor 77 (GPR-77) and are proficient in promoting CSCs and their stemness properties and inducing chemoresistance of tumor cells through persistent NF-κB activation along with the resultant IL-6 and IL-8 secretion (72). Of note, these pro-stemness subset of CAFs were either defined by surface markers (e.g., CD10 and GPR-77), their transcriptome and secretome (e.g., CAF-D), or a specific set of secretory factors (e.g., IL-6 and LIF in iCAFs). It remains to be established if the pro-stemness subset of CAFs indeed vary among different types of cancers or can be molecularly defined in a more precise manner.

In chemotherapy-treated desmoplastic cancers, CAFs are endowed with additional pro-stemness and pro-oncogenic capabilities as a result of the stress-induced chronic phenotypic and functional alterations. For instance, in prostate cancer, the genotoxic agent mitoxantrone stimulated Wnt-16B secretion by stromal fibroblasts, which promoted the proliferation and invasion of carcinoma cells, which likely contained the enriched CSCs (53). In human CRC, chemotherapy led to the enrichment of IL-17A-producing CAFs within the tumor stroma, which in turn promoted the self-renewal of CSCs and tumor growth (36). In addition, following systemic chemotherapy, breast and pancreatic CAFs secreted large amounts of the “ELR-motif-positive” (ELR^+^) CXCL chemokines through chronic activation of the STAT-1 and NF-κB transcriptional activities, which stimulated CXCR-2 signaling in cancer cells to elicit their transdifferentiation into CSCs and thereby promoted post-treatment tumor aggression and treatment failure (37).

Despite the multiple lines of evidence supporting the pro-stemness functions of CAFs and therapy-modulated CAFs, it is worth noting that, as highlighted above, CAFs are capable of promoting tumor progression and treatment resistance through a multitude of mechanisms. Therefore, the tumor-promoting effects of CAFs summarized herein may likely be mediated by the concerted actions of a plurality of mechanisms and should not be attributed only to the pro-stemness functions of CAFs. Moreover, since most of these studies were conducted in immuno-compromised or immune-deficient animal models, caution should be exercised while interpreting the results related to the CAF-derived pro-stemness cytokines and chemokines that are actively involved in inflammation and immune regulation. Whether or not CAFs exert similar positive regulatory effects on CSCs in immunocompetent backgrounds and how these effects work in concert with other tumor-promoting mechanisms of CAFs await further in-depth investigation.

## Tumor-Infiltrating MSCs and Their Interplay With CSCs

MSCs were initially considered to be cells promoting the regenerative properties of wounds and damaged tissues. A growing body of evidence indicated that the regenerative function of MSCs are hijacked by malignant tumors such that a significant number of bone marrow-derived MSCs are recruited to the tumor microenvironment, where a considerable proportion of them differentiate into CAFs (40, 41). Like CAFs, MSCs can secrete a plethora of cytokines and growth factors, which make them proficient in paracrine and heterotypic signaling processes. For instance, a recent comprehensive cytokine secretion profile of human MSCs identified IL-6, IL-8, TIMP metallopeptidase inhibitor 2 (TIMP-2), CCL-2 (MCP-1), and vascular endothelial growth factor (VEGF) as the most abundantly secreted factors (82). Other studies reported that MSCs promote cancer metastasis by secreting CCL-5 (15), CXCL-12, and IGF-1 (83). MSCs also contribute to tumor angiogenesis by secreting vascular endothelial growth factor (VEGF), and β-fibroblast growth factor (FGF) (67). Furthermore, MSCs promote immunomodulation by upregulating cytokines such as IL-6, IL-8, and TGF-β (68, 69, 84). In breast cancer, the cancer cells stimulate the secretion of CCL-5 from MSCs, which acts in a paracrine fashion on the cancer cells to enhance their motility, invasion, and metastasis (15). Importantly, several studies have directly implicated MSCs in the regulation of CSCs (Figure 1). MSCs in breast cancer regulate CSCs through cytokine loops involving IL-6 and CXCL-7, thereby accelerating tumor growth (44). In CRC, MSCs secrete prostaglandin E2 (PGE-2) in response to IL-1 released by carcinoma cells, which act in an autocrine fashion to induce the expression of IL-6, IL-8 and CXCL-1, which together induce the formation of CSCs (46). Once differentiated into CAFs, MSCs can maintain CSCs through secreting the Notch ligand Jagged-1 (47). In analogous to the effect of chemotherapy on the number and the pro-stemness property of CAFs, the number of bone marrow-derived MSCs significantly increased following gemcitabine treatment in the tumor stroma in a mouse xenograft model of PDAC (66). Importantly, these gemcitabine-educated MSCs were found to have a positive regulatory effect on CSCs through the STAT-3–CXCL-10–CXCR-3 paracrine signaling axis. Similarly, following paclitaxel treatment or hyperthermia therapy, MSCs secreted IL-6, IL-7, IL-8, EGF, and IGF, which supported drug resistance (85, 86). In another study, cisplatin-activated MSCs produced specific polyunsaturated fatty acids which in turn promoted the regrowth of tumors following therapy (70).

Collectively, the ample evidence underscore the important role of CAFs and tumor-infiltrating MSCs in the maintenance and the expansion of CSCs and suggest that targeting this component of the tumor stroma may provide a new avenue to improving the therapeutic outcome of human desmoplastic cancers.

## Targeting the Crosstalk Between CAFs and CSCs

**Figure 2:**
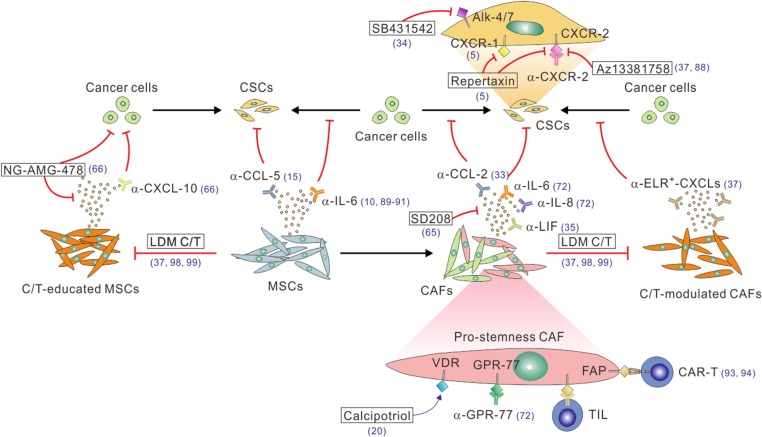
A multitude of approaches to block the CAF/MSC–CSC crosstalk. Function-blocking antibodies, including α-IL-6, α-IL-8, α-LIF, α-CCL-2, and α-CCL5, or small molecular inhibitors, such as the TGF-β inhibitor SD208, can be used to block the stimulatory effect of these pro-stemness factors secreted by CAFs or C/T-modulated CAFs. On the other hand, the CAF-CSC paracrine signaling can be blocked by function-blocking antibodies (e.g., α-CXCR-1, α-CXCR-2) or small molecule inhibitors (e.g., repertaxin, AZ13381758, SB431542) of the receptors on CSCs and/or cancer cells. Likewise, the pro-stemness functions of tumor-infiltrating MSCs can be antagonized by function-blocking antibodies against IL-6 or CCL-5. The enhanced pro-stemness functions of C/T-modulated CAFs or MSCs can be potentially blocked by function-blocking anti-ELR^+^-CXCL-chemokine antibodies, anti-CXCL-10 antibody, the CXCL-10 inhibitor AMG-478 (encapsulated by MSC-derived nano-ghost, NG), or adopting low-dose metronomic (LDM) C/T regimens. Function-blocking α-GRP-77 antibodies can be used to reduce the tumor infiltration of CD10^+^GPR-77^+^ pro-stemness CAFs. Calcipotriol can activate VDR signaling to inhibit IL-6, CCL-2, and CXCL-1 production by CAFs. Finally, FAP^+^ or GPR-77^+^ CAFs can be depleted by using DNA vaccines to induce CAF-specific tumor-infiltrating T cells (TILs) or administrating CAF-specific CAR-T cell or other types of engineered immune cells. The reference numbers are shown in blue.

Given that CAFs positively regulate CSCs through the secretion of pro-stemness paracrine factors, a number of preclinical studies have exploited the therapeutic potential of the functional blockade of the CAF-to-CSC paracrine signaling process to improve the treatment of desmoplastic cancers (Figure 2). For instance, loss of PTEN in a HER2-overexpression genetic background or the trastuzumab resistance in breast cancer cells has been linked to activation of the IL-6/STAT-3/NF-κB inflammatory loop, which induced an EMT phenotype and expansion of the CSC population. Therefore, blocking this loop by a function-blocking anti-IL-6 receptor antibody could effectively revert these phenotypes (10). In another study, functional inhibition of the subgroup of IL-6- and IL-8-secreting CD10^+^GPR-77^+^ CAFs with anti-IL-6 and anti-IL-8 antibodies, together with docetaxel chemotherapy, has led to a near complete remission of tumors in a patient-derived xenograft (PDX) model of breast cancer (72). Interestingly, this study also demonstrated that an anti-GPR-77 antibody in combination with docetaxel therapy exerted anti-tumor efficacy comparable to that induced by the combination anti-IL-6/anti-IL-8 therapy, which significantly reduced the number of CD10^+^GPR-77^+^ CAFs and the proportion of CSCs in the treated tumors (72). In keeping with the critical role of IL-8 in cancer stemness, a function-blocking antibody against its receptor, CXCR-1, or a small-molecule inhibitor of CXCR-1 and CXCR-2, repertaxin, could deplete CSCs and inhibit tumor aggressiveness in human breast cancer xenografts (5). Of particular clinical relevance was the recent finding that repertaxin in combination with paclitaxel demonstrated a 30% response rate in a phase 1b study of metastatic breast cancer (87). Other small-molecule inhibitors of CXCR-2, including AZ13381758 and SB225002, have also shown preclinical efficacy in transgenic or PDX models of PDAC (37, 88). Of note, since that CXCR-2 is also expressed by myeloid-derived immunosuppressive cells in PDAC (88), its inhibitor may exert anti-tumor efficacy through multiple mechanisms of action. Aside from the molecular targeting of IL-6, IL-8 and their receptors, targeting their downstream signaling components in cancer cells and/or CSCs offer other viable opportunities for disabling the CAF–CSC crosstalk. For instance, a small-molecule inhibitor of STAT-3, BBI608, has been reported to significantly inhibit cancer stemness in a variety of cancer types (89), whereas the results from some of the recent clinical trials were discouraging. Furthermore, several novel therapeutics targeting the CAF-to-CSC IL-6–STAT-3 signaling axis are under development. These include a high-affinity anti-IL-6 antibody, MEDI5117, which has been shown to enhance the anti-tumor efficacy of chemotherapy or gefitinib in several types of tumors that are known to be driven by the IL-6–STAT-3 signaling and especially target the CD44^+^CD24^−^ CSCs in trastuzumab-resistant and HER-2^+^ breast cancer cells (90). Another example is a cyclic oligonucleotide decoy that corresponds to the STAT-3 response element of STAT-3-targeted genes, which showed promising anti-tumor efficacy in NSCLC models (91). Recently, another interleukin family protein, LIF, was found to be the major PSC-derived factors that promote CSCs in PDAC cells and tissues (35). Accordingly, systemic administration of a LIF-neutralizing antibody in combination with chemotherapy reduced the percentage of CSCs and mesenchymal-transited cancer cells and extended the survival of tumor-bearing mice in a transgenic model of PDAC.

Aside from inhibiting interleukin paracrine signaling, a number of studies have explored the therapeutic potential of inhibiting other CAF-derived pro-stemness factors. For instance, the CCL-2 neutralizing antibody has been shown to significantly suppress tumorigenesis and inhibit pro-stemness Notch signaling in an orthotopic breast cancer model involving the co-implantation of cancer cells and CAFs (33). The TGF-β inhibitor SD208 has been shown to reduce the CAF-induced expression of stemness markers and simultaneously induced the expression of differentiation markers in CAF-cocultivated CRC cells (65). Consequently, SD208 in combination with the small molecule inhibitor of GLI-2, a transcriptional factor in the SHH pathway, restored the sensitivity of the tumors to chemotherapy in mouse PDX models of CRC. SB431542, an inhibitor of the Nodal receptor Alk-4/7 expressed on pancreatic CSCs, could block the stemness and invasive capacities of CSCs and thereby inhibited PDAC progression especially when used in conjunction with a Smoothened (a SHH pathway receptor) inhibitor that depleted the tumor stroma (34). Nevertheless, it is worthy of note that these promising results should be interpreted with caution since most of the studies were conducted in immuno-deficient mice without the potential influence from the immune system, which is especially relevant as most of the pro-stemness mediators studied also have pro-inflammatory and immune-related functions.

Given the profound impacts of CAFs on the tumor stemness and aggressiveness in desmoplastic cancers, depleting CAFs in the tumor stroma provided another viable option in attenuating the CAF–CSC interplay (Figure 2). Along this line, an oral DNA vaccine targeting FAP, a CAF-specific marker, has been demonstrated to suppress tumor growth and metastasis and confer a survival benefit in murine models of CRC and breast cancer (92). Moreover, adoptive transfer of FAP-targeted chimeric antigen receptor (CAR) T cells could specifically kill FAP^+^ CAFs and induce multiple beneficial stroma alterations, leading to delayed tumor growth and survival extension in mouse models of NSCLC and PDAC (93, 94). Interestingly, a combined targeting of FAP^+^ CAFs and EPH receptor A2 (EphA2)^+^ cancer cells led to a nearly complete remission of the tumors (93), suggesting that CAF-targeted approaches have the potential to supplement and synergize with conventional cancer-cell-targeted therapies. Notwithstanding these promising results, caution must be exercised with the application of CAF-depleting strategy as the genetic depletion of CAFs using the conditional knockout of SHH signaling or the ganciclovir-induced depletion of CAFs in PDAC unexpectedly led to invasive and undifferentiated tumors along with unfavorable immunosuppression (79, 95). Furthermore, depletion of FAP^+^ stromal cells in a transgenic mouse model has been associated with muscle wasting and impaired erythropoiesis (96), implicating the potential adverse effects of CAF-depleting therapies. In this regard, the functional inhibition of CAFs instead of their depletion may be a safer and more desirable therapeutic approach than the direct depletion of CAFs. Several recent studies toward this direction have shown promises. For instance, vitamin D receptor (VDR) signaling has been shown to antagonize TGF-β/SMAD signaling-induced activation of PSCs in PDAC tissues, which was mediated by the pro-stemness factors IL-6, CCL-2, and CXCL-1 (20). As such, calcipotriol, a potent vitamin D analog that controls VDR induction, inhibited inflammatory signaling in CAFs and reduced the expressions of IL-6, CCL-2, and CXCL-1. When combined with gemcitabine treatment, calcipotriol synergized with chemotherapy to control tumor growth and extend survival in transgenic mouse models of PDAC. Another approach involved transducing CAFs with a nanocarrier-formulated plasmid encoding a secretable form of the death ligand TNF-related apoptosis-inducing ligand (TRAIL) termed sTRAIL. Since CAFs are intrinsically resistant the killing effect of TRAIL, once transduced with the sTRAIL-expressing plasmid, they were converted into sTRAIL-producing cells and thereby triggered apoptosis of neighboring cancer cells (97). Surprisingly, the expression of sTRAIL also reprogrammed CAFs into a quiescent state. This approach demonstrated strong anti-tumor efficacy in a PDAC model.

Recently, low-dose metronomic (LDM) chemotherapy has emerged as a highly clinical applicable strategy to enhance the tumoral treatment response by tempering the therapy-induced stromal alterations in desmoplastic cancers (37, 98, 99). Compelling evidence from laboratory-based and clinical correlative studies have demonstrated that conventional chemotherapy administered at a maximum tolerated dose (MTD) induces myriad alterations in stromal cells, including endothelial cells and their progenitor cells, immune cells, and CAFs (53, 100). In keeping with this paradigm, our group recently demonstrated that systemic MTD chemotherapy of assorted agents, including paclitaxel, gemcitabine, doxorubicin, and cyclophosphamide, had profound impacts on CAFs in human breast cancer and PDAC tissues, which acquired the ability to secret large amounts of pro-stemness ELR^+^ CXCL chemokines through the chronic activation of STAT-1 and NF-κB signaling (37). The CSC niche microenvironment generated by therapy-modulated CAFs could be attenuated by pretreating the tumors with a CXCR-2 inhibitor or by switching the dosing schedule to LDM regimens, which had a much less stimulatory effect on CAFs. We envisage that this approach has multiple benefits. First, it obviates the lengthy and costly process of developing new CAF- and/or CSC-targeted agents, which has an especially high attrition rate according to past experiences. Second, an increasing number of oral chemotherapeutic agents are available for clinical use, making the concept of LDM chemotherapy immediately clinical applicable. Third, as mentioned earlier, LDM chemotherapy not only may prevent the CAF–CSC interplay but may also exert multiple favorable effects on other cells in the tumor stroma, including tumor-associated macrophages, myeloid-derived suppressor cells, and blood vessel cells (98, 99, 101, 102). We thus foresee that LDM chemotherapy will become the treatment of choice in many types of desmoplastic cancers.

## Targeting the Crosstalk Between MSCs and CSCs

Due to their pro-tumorigenic activities, a number of studies had been carried out to try and target MSCs as a therapeutic approach in cancer (103). Specifically, given that tumor-infiltrating MSCs can directly support CSCs through multiple paracrine signaling pathways, including IL-6, IL-7, CXCL-1, PGE-2, Jagged-1, and CXCL-10 (44, 46, 47, 66), blockade of the molecular crosstalk between MSCs and CSCs may be potentially useful in inhibiting cancer stemness in desmoplastic cancers. Indeed, a recently study exemplified the potential utility of this approach (66). In a mouse model of PDAC, MSCs were found in close proximity to CSCs following gemcitabine chemotherapy, implicating MSCs as the CSC niche. Mechanistically, gemcitabine-exposed MSCs secrete high levels of CXCL-10 that activate its receptor CXCR-3 on CSCs, activating STAT-3 signaling and promoting the survival of CSCs. Consistently, systemic administration of the CXCL-10 inhibitor AMG487 formulated with MSC-derived membrane-based nanoparticles termed “nano-ghost (NG)” led to its intratumoral accumulation in close proximity to CSCs, thereby reducing the percentage of CSCs and augmenting the therapeutic efficacy of gemcitabine. In analogous to the directly depletion of CAFs, the direct depletion of MSCs might provide an alternative approach to nullify their crosstalk with CSCs. However, whether MSC-deprived host for a limited time may cause toxicity on its own remains an open question. Alternatively, given that MSCs secrete CXCL-10 in response to gemcitabine treatments, and that gemcitabine given at an LDM regimen could attenuate therapy-induced production of pro-stemness chemokines from CAFs in PDAC (37), it is highly likely that LDM chemotherapy may also prevent or at least attenuate chemotherapy-induced activation of MSCs and their secretion of pro-stemness chemokines. This possibility merits further investigations. On the other hand, since MSCs secrete the pro-stemness cytokine IL-6 (46), the various anti-IL-6 antibodies and/or STAT-3 inhibitors developed to inhibit the CAF–CSC crosstalk can also be adopted to block the interaction between MSCs and CSCs (10, 89–91). This raises the possibility that MSC- and CAF-targeted therapeutics may synergize with each other. We thus envisage that the CAFs and MSCs dual targeting approach may provide an opportunity to more thoroughly block the stroma-derived pro-stemness signals to maximize the anti-tumor efficacy in the treatment of desmoplastic cancers.

## The Unique Advantages of Targeting Pro-Stemness CAFs and MSCs

As opposed to the direct targeting of CSCs, which poses significant challenges, targeting CAFs or MSCs along with the pro-stemness niches they generate may have several advantages in the treatment of desmoplastic cancers (Table 2). First and foremost, a growing body of evidence now suggests that CSCs are highly heterogeneous and plastic and the conversion between different CSC populations plays an important role in tumor progression and treatment response (104). For instance, breast cancer CSCs exist in alternative mesenchymal-like and epithelial-like states which can transition between each other (105–107). CSCs can also be derived from differentiated cancer cells through cellular reprogramming or transdifferentiation (11), which can be particularly facilitated by cytotoxic stresses such as chemotherapy and ionizing radiation (37, 108). The highly dynamic nature of CSCs makes them moving targets in cancer therapy, which presents a daunting challenge to therapeutic efforts aiming at completely eradicating them. Echoing this notion, two recent studies in the organoid models of CRC highlighted the difficulty of eradicating CSCs. Specifically, ablation of LGR-5^+^ CSCs halted tumor growth, whereas the tumors resumed growth following the removal of the cell death inducers due to the re-emergence of CSCs from differentiated tumor cells (109, 110). As a comparison, CAFs are both genetically and phenotypically stable; therefore, CAF-directed therapies may lead to a more stable and sustainable anti-CSC effect compared with that results from the direct targeting of CSCs. Second, the recent discoveries of specific subpopulations of pro-stemness CAFs have rendered CAF-directed therapy more feasible as they not only provide novel therapeutic targets, such as GPR-77 (72), but also rendered the related therapies more specific and safer than the non-specific targeting of CAFs (79). Another unique advantage of targeting pro-stemness CAFs relates to their spatial distributions within desmoplastic cancers. Specifically, CAFs and tumor-infiltrating MSCs exist in large numbers in the tumor stroma, which contrasts sharply with CSCs that comprise only a small or even a rare subpopulation of cancer cells and exist within cancer cell nests or as individually dispersed cells or small cell clusters at the tumor periphery or the invasive front (111, 112). In desmoplastic cancers such as PDAC, there are abundant CAFs in the stroma, which can account for more than 90% of the total tumor volume (113, 114). Thus, there are a far larger number of CAFs or MSCs that can be exposed to the therapeutics administrated at a given tissue concentration than that of CSCs. Accordingly, CAF- or MSC-targeted therapeutics may have better pharmacodynamic effects than CSC-targeted agents in the treatment of desmoplastic cancers. Moreover, CAFs are often localized to the periphery of the tumor cell nests or glands and close to blood vessels, rendering them directly accessible to the therapeutics diffused from the blood circulation (115). By contrast, carcinoma cells, including the small population of CSCs, are frequently distantly spaced from blood vessels in desmoplastic tumors. In fact, CAFs *per se* constitute a significant barrier for the therapeutic delivery of drugs and even nanoparticles to cancer cells (97, 116). Echoing the importance of the spatial distribution of cells in the treatment of poorly perfused desmoplastic tumors, clinical data has confirmed that the majority of therapeutics, such as gemcitabine, can only reach the stroma of human PDAC tissues (117). Collectively, these factors make targeting the link between CAFs or MSCs with CSCs more justified, feasible and clinically promising than the direct targeting of CSCs in the treatment of desmoplastic cancers.

**Table 2:** The potential advantages of targeting pro-stemness CAFs and MSCs.

**Characteristics**	**CAFs or MSCs**	**CSCs**	**Advantages of CAF targeting**
Genotype	Relatively stable	Heterogeneous	More constant effects and less treatment failure
Phenotype	Relatively stable	Highly dynamic and plastic	
Density in tumor	High (especially in desmoplastic cancer)	Rare to low	Favorable pharmacodynamic effects
Localization in tumor	Tumor periphery or surrounding blood vessels	Within tumor cell nests or at the invasive front	More accessible to therapeutics

## Biomarkers of Pro-Stemness CAFs

Tumor cells are highly heterogeneous in terms of their phenotypes, genotypes, and functions. As aforementioned, it is increasingly recognized that the intra-tumoral heterogeneity not only exists in the epithelial compartment but also the stromal compartment of the tumors, including CAFs (29, 71, 72). As such, human desmoplastic cancers may vary considerably with respect to the number as well as the composition of CAFs, including those with pro-stemness properties. Clinical trials investigating therapies targeting the CAF-to-CSC crosstalk should be ideally conducted in a patient- and tumor-tailored manner based on surrogate markers of CAF activation and/or their pro-stemness functions. We list a number of CAF-related biomarkers that may potentially fulfill this purpose (Table 3). First, a high density of α-SMA^+^ CAFs in tumors has been linked to the resistance to neoadjuvant chemotherapy in breast cancer (72). Therefore, the density of CAFs may serve as a simple and immediately clinically applicable biomarker based on which CAF-targeted therapies can be implemented. Likewise, the density of CAFs also significantly increased following systemic chemotherapy in human CRC tissues (36). A plausible corollary is that the density of CAFs positively correlates with the likelihood of treatment resistance in most desmoplastic cancer and thus can serve as a universal biomarker to guide CAF-targeted therapies. Notably, since different CAF markers, including such as α-SMA, FAP, and FSP-1, may identify functionally distinct CAF populations that vary among different cancer types of subtypes (76, 77, 79), it remains to be established which CAF marker or any of their combinations can serve as a clinically informed biomarker. Beyond simply measuring the density of CAFs, the staining intensity of phosphorylated STAT-1 in CAFs, which reflects their ability to produce pro-stemness chemokines following chemotherapy (37), may also aid the clinical decision-making regarding when CAF-directed therapies should be implemented. On the other hand, in untreated tumors, the density of pro-stemness CAFs, such as α-SMA^−^PDGF-Rα^+^IL-6^+^ iCAFs in PDAC and CD10^+^GPR-77^+^ CAFs in breast cancer and NSCLC (71, 72), can serve as a companion diagnostic to guide the selection of patients for anti-CAF/CSC therapies, especially those targeting the IL-6 and/or the IL-8 paracrine signaling pathways. In theory, these CAF-related biomarkers can be further combined with widely used surrogate markers of CSCs, such as ALDH, CD133, CD44, CD24, CD90, and EpCAM (118, 119), to increase their predictive power and clinical utility. We predict that the application of these CAF-related stemness markers may increase the success rate of the related clinical trials and pave the road for the next-generation patient-tailored anti-cancer therapies.

**Table 3:** Biomarkers linked to pro-stemness CAFs that can guide clinical studies.

**Biomarker**	**Significance**	**Clinical setting**	**Cancer type**	**References**
CAF density^a^	A high density of CAFs is associated with resistance to chemotherapy	Adjuvant or combination therapy	BC, CRC	(31, 62)
Phosphorylated STAT-1^+^ fibroblasts	Positive staining indicates pro-CSC CAFs following chemotherapy	Adjuvant or combination therapy	BC, PDAC	(32)
SMA^−^PDGF-Rα^+^IL-6^+^ fibroblasts	Reflects the number of pro-CSC CAFs in breast cancer or NSCLC	Neoadjuvant or combination therapy	PDAC	(61)
CD10^+^GPR-77^+^ fibroblasts	Reflects the number of pro-CSC CAFs in PDAC	Neoadjuvant or combination therapy	BC, NSCLC	(62)
ALDH^+^, CD133^+^, CD44^+^, CD24^+^, CD90^+^ and/or EpCAM^+^ cancer cells^b^	Reflects the density of CSCs	Neoadjuvant or combination therapy	When applicable	(108, 109)

*BC, breast cancer; CRC, colorectal cancer; PDAC, pancreatic ductal adenocarcinoma; NSCLC, non-small cell lung cancer*.

a *Identified using reported CAF markers, including FAP, α-SMA, FSP-1, PDGFR-α/β, etc, or their combinations*.

b *Used in combination with CAF-related markers*.

## Important Considerations and Potential Challenges in the Clinical Development of Pro-Stemness-CAF- or MSC-targeted Therapies

Whilst targeting pro-stemness CAFs and MSCs have multiple theoretical advantages over the direct targeting of CSCs, several potential challenges remain and require careful considerations at the various developmental stages of the therapies. First, since CAFs or MSCs maintain their crosstalk with CSCs mainly through pro-stemness cytokines and chemokines, the majority of CAF- or MSC-targeted therapeutics are function-blocking antibodies (Figure 2). It is widely accepted that large-molecule therapeutics like antibodies have very limited penetration into desmoplastic tissues and may only be able to reach CAFs or MSCs spaced at the outer rim of tumors or those located surrounding or near blood vessels. If so, their anti-CSC and anti-tumor efficacy will be severely compromised (120). One potential solution for this problem is pre-treating desmoplastic tumors with agents that can reduce the number of CAFs and/or the desmoplastic reaction they produce, which can be exemplified by the stroma-reducing effect of *nab*-paclitaxel and SHH inhibitors in human PDAC (34, 117). Another solution is by using small-molecule inhibitors or nanoparticles designed to block pro-stemness factors or their receptors, such as repertaxin, SD208, BBI608, calcipotriol, and NG-AMG487, which have the ability to diffuse deeply into the desmoplastic stroma and reach their intended target cells compared with antibodies. Second, CSC-directed therapies, which target only a small subpopulation of cancer cells, would not be expected to produce measurable changes in tumor burden according to conventional Response Evaluation Criteria in Solid Tumors (RECIST) criteria. Therefore, more pertinent, “stemness-informed” surrogate markers of response that are applicable to anti-CSC agents should be developed to guide the conduction of clinical trials, especially at the phase II stage (121). This concern should be also taken into consideration when conducting clinical trial testing CAF- or MSC-targeted therapies designed to specifically target CSCs. We propose that this problem can be at least partially tackled by introducing stemness-informed CAF- or MSC-related biomarkers as described in Table 2. Third, as described above, CAFs or MSCs are spaced in the tumor stroma, whereas CSCs exist mainly within tumor nests or as individually dispersed cells or small cell clusters at the tumor periphery or the invasive front. Therefore, a plausible corollary is that the CAF/MSC–CSC crosstalk through paracrine signaling will predominantly take place at the tumor periphery. If so, pathological biomarkers and criteria that reflect the distance between CAFs or MSCs and CSCs should be developed to select those tumors that most likely respond to therapies directed at disrupting the CAF/MSC–CSC interplay. Finally, the timing of implementing pro-stemness-CAF- or MSC-targeted therapies will be another important consideration in the design of the related clinical trials. For the agents designed to target treatment-naïve CAFs or MSCs, it is critical to dose patients in early phases of cancer treatment before or concurrently with neoadjuvant chemotherapy as CSCs are less frequent and may be more susceptible to CSC-directed agents (54). By contrast, for the therapeutics targeting chemotherapy-modulated CAFs or MSCs, they should be administered following the initiation or during the course of chemotherapy, depending on when and the extent to which the pro-stemness functions of CAFs or MSCs are activated. Another timing of CAF/MSC-CSC-directed therapy is at the adjuvant setting following the removal of primary tumors, at which the therapy is designed to target micro-metastatic and circulating tumor cells that are known to contain enriched CSC populations (122–124). In this scenario, the blockade of CAF- or MSC-derived pro-stemness factors is expected to prevent the formation of CSC niches in primary or distant sites to reduce tumor recurrence and/or metastasis following surgery. Again, appropriate stemness- and/or stroma-informed biomarkers will be required to guide patient selection as well as the prediction of response in this type of trial.

## Conclusions and Future Directions

The first generation of therapeutic strategies aiming at blocking the CAF-derived pro-stemness factors has remained largely in preclinical stages or been tested in early-phase clinical trials. Further optimization and improvements in the potency of antibody and small-molecule therapeutics or the introduction of novel therapeutic entities, such as the STAT-3-targeted oligonucleotide (91), may hold promises to overcoming current developmental hurdles. Alternatively, functional targeting or the specific depletion of the pro-stemness subpopulation of CAFs using such as FAP- or GPR-77-targeted antibodies, DNA vaccine, and immune cell therapeutics, provides promising next-generation approaches to preventing the cross-talk between CAFs and CSCs. Suppressing the pro-stemness factors secreted by MSCs or the direct depletion of MSCs also represents an interesting and promising opportunity of antagonizing their pro-oncogenic effects. The pro-stemness-CAF- or MSC-targeted therapies offer a novel opportunity of enhancing the treatment response of cytotoxic therapies such as chemotherapy and IR to prevent treatment-triggered expansion and activation of CSCs. Moreover, pro-stemness-CAF- and MSC-targeted therapies may synergize with CSC-targeted agents to reduce cancer stemness and aggressiveness, ultimately improving the therapeutic outcome of patients with desmoplastic cancers. Pro-stemness-CAF-related biomarkers are expected to aid the design of clinical trials and guide patient selection in CAF-/MSC-targeted therapies. Whilst these novel stroma-targeted approaches may potentially renew the interest in CSC-directed therapies in solid tumors, whether or not they can indeed fulfill their promise remains to be validated by more meticulously designed clinical trials.

## Author Contributions

T-SC: conceptualization, drafting of the manuscript, and critical revision of the manuscript. YS: drafting of the manuscript. KT: conceptualization, drafting of the manuscript, critical revision of the manuscript, obtained funding, final supervision, review, and editing.

### Conflict of Interest Statement

The authors declare that the research was conducted in the absence of any commercial or financial relationships that could be construed as a potential conflict of interest.
